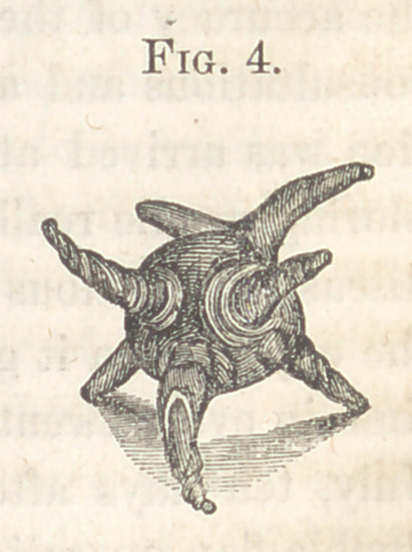# Proceedings of the Pathological Society of Philadelphia

**Published:** 1858-03

**Authors:** 


### ART. VII.—*Proceedings of the Pathological Society of Philadelphia.*

Reported by the SECRETARY.

WEDNESDAY EVENING, NOV. 25th, 1857.

The President in the chair.

*Eburnation of Bone.*—DR. PACKARD called the attention of the Society to a specimen of disease of the hip and knee-joints, obtained in the University dissecting room several years ago, from a male subject about fifty years of age; no history of the case could be procured.

The right acetabulum is seen to be enlarged, and the surface of the neighboring bony tissue roughened by adventitious deposit. The upper part of its edge is irregularly absorbed to some extent; at the upper and back part of the cavity the surface is roughened, and of a worm-eaten appearance, to the extent of $1\frac{1}{4}$ by 2 inches. Just at the edges of this patch the bone is eburnated, being very hard, white, and polished. The foveola is nearly filled with bony deposit.

The head of the femur has exactly the same characters as the acetabulum at precisely corresponding points, except at the part opposite the foveola, where it is unaltered. The *corona*, if it may be so called, and the entire cervix of the bone, are covered with a very thick and irregular adventitious deposit of bony matter. Both trochanters, the inter-trochanteric lines, and the linea aspera, are also thickened and roughened.

The left knee-joint seems healthy, except that on the articular face of the inner condyle of the femur is a patch $1\frac{1}{4}$ inch wide by $2\frac{3}{8}$ inches long, which is very exquisitely polished, evidently by friction upon the corresponding surface of the tibia, where a similar patch is seen, differing only that it is $1\frac{1}{2}$ inch wide by $1\frac{5}{8}$ long. Around the articulating extremities of the femur, tibia, and fibula are irregular adventitious deposits of bony matter.

The other hip and knee-joints were diseased in the same way, but to a much less degree; they were not preserved.

This would seem to have been an instance of the affection described by R. W. Smith, in his work on *Fractures and Dislocations*, under the name of "Chronic Rheumatic Arthritis." Mr. Benjamin Bell calls it "Interstitial Absorption of the neck of the thigh-bone." Dr. Packard believed that it was rarely seen in the knee-joint so well characterized as in this specimen.

*Cystic Tumors on the Nates, obstructing Delivery.*—DR. KELLER exhibited two remarkable specimens of cysts taken from the nates of children, and which had presented a great obstacle to their delivery. The following histories of them were reported:—

CASE I.—Mrs. R., about twenty-five years old, had previously been delivered of two full-grown, healthy children. She was at the time in very good health, and the prospects for a third easy delivery were very favorable, the child being found with the vertex presenting. After a few pains the head was born, and the trunk of the child followed easily up to the hips; here it stopped; and when the attending physician tried to extract it in the usual way, he found an obstacle which he could not overcome even by exerting all his force. Having called in some experienced colleagues, the blunthook was used, by which the child was soon delivered, alive, but unfortunately with the left thigh broken by the application of this dangerous instrument. Owing to this accident, and to a deformity on the back, the physicians declared the child could not live. Three days after the delivery of the woman, (on the 6th of December, 1855,) I was called to see the child in consultation, the mother being sick and the child in a desperate state, as the fractured leg had begun to mortify. When I examined the child I found a tumor, (see Fig. 1,) of the size of a man's head, attached to the nates, exactly behind the orificium ani, and containing a liquid. On the right side, at the base of the tumor, could be felt, by a careful examination and strong pressure through the liquid, another hard tumor, in form and size like an egg. The child not being expected to survive, had not received any nourishment excepting a little tea and water. In order to settle the question whether the child could live or not, it was proposed, on the eighth day, to empty the sac by a puncture. More than a quart of a brownish liquid escaped, which became of a bloody color as it continued to flow. The child died a few hours afterwards, from a hemorrhage into the sac. A post-mortem examination was performed the next day. The viscera were all found in a perfectly healthy state, and did not communicate with the tumor. The sac was shrivelled into folds, and contained a large clot of blood. It was a perfect cyst, the parietes of which were very dense, covered in some parts, particularly at its insertion into the nates, with soft conglomerations of blood-vessels. The smaller, hard tumor above mentioned, which was felt inside of the sac, was formed by cysts, having dense but transparent membranes, and containing a clear liquid. At no point did the cyst communicate with the spinal column.

FIG. 1.

CASE II.—On the 21st of March, 1856, at 2 o'clock A.M., I was called to attend Mrs. T., in labor; I found the patient in a strange situation, as the child, having presented in a position of the vertex, had made its full exit, but could not be separated from the mother, being attached by a pedicle to a large body remaining in the womb, which was supposed to be another child. Before examining, I suspected a cystic tumor, and in this opinion was fully confirmed by a close examination. I immediately punctured the tumor with a curved trochar, and after the evacuation of about a gallon of a brownish liquid the sac was emptied, and the child naturally expelled. It died about six hours after its delivery by a capillary hemorrhage into the sac. The liquid unfortunately could not be examined chemically, as it was thrown away in spite of my strict injunction. The sac showed externally the natural appearance of the skin; the walls were about one-sixth of an inch thick. The internal surface was covered on the upper part with a soft tissue, which showed under the microscope a rich network of capillary blood-vessels, very similar to the villi of the intestines. The lower part was covered with a reticulated fibrous tissue. The tumor was inserted between the orifice of the rectum and the point of the coccyx, as in the first case; not exactly in the centre, but about two inches to the right side. A careful examination proved that the tumor did not communicate with the vertebral canal.

*Fœtal Monstrosity.*—DR. HEWSON exhibited the head and neck of a fœtal calf, presenting four eyes, two jaws. The skulls are blended; there are four lobes of cerebrum and cerebellum, each distinct; the medulla is single; the tongue bifid; the larynx and trachea single; the decussation of the optic nerves obvious; articulation of the jaws blended, but movable; sclerotic of the contiguous eyes blended.

*Fracture of Cervical Vertebræ.*—DR. MORTON exhibited several fractured cervical vertebræ taken from a young man twenty-three years of age, who fell down the hold of a vessel and struck with violence the back of his neck. He lived eleven days, then died of suffocation. The body was paralyzed from the arms down; the patient could lift his arms, but could not flex or extend the fingers. A marked symptom was retention of urine, which lasted until within two days of death, when the urine passed involuntarily from him. The heat of surface two hours after the accident was 102° Fahr. On post-mortem examination the transverse process of the fifth vertebra was found to be broken, and pressing on the spinal cord. The spinous process of the sixth was broken off; also a considerable portion of the bony bridges which connect the spinous with the oblique processes were fractured and pressed downward, but did not touch the cord. The bodies of the sixth and seventh vertebræ were crushed one into the other.

*Laceration and Severance of the Cervix Uteri.*—DR. KELLER presented a specimen of ruptured neck of the uterus. When the patient was examined by him, during labor, the lips of the uterus were much swelled and enlarged; the os tincæ admitted two fingers. The head presented; the womb was in a position of anterior obliquity. The head of the child pressed on the posterior wall and lip of the womb, which it finally ruptured. The child was still-born, after several hours' labor, and a tumor was then found protruding beyond the external parts. A ligature was applied around its base, and the tumor came away five days afterwards. It is now two months after the accident, and the patient is well. Indeed, at no time did she present any unfavorable symptoms. On examining the tumor carefully, it was found that it consisted of the healthy neck of the uterus, which had been severed and carried down by the child's head.

*Deformity of the Neck of the Thigh-Bone, simulating Fracture with Ossific Union.*—DR. RICHARDSON exhibited a specimen (see Fig. 2) of deformity of the neck and head of the femur, for the purpose of calling attention to the liability of mistaking such deformities for ossific union of fracture of the bone within the capsular ligament. The specimen was recently obtained from the body of a man, apparently about twenty-five or thirty years of age, and of good physical development, brought into the dissecting room of the Medical Department of Pennsylvania College; and upon first inspecting it Dr. Richardson supposed it possible that a fracture of the cervix, followed by bony union, might have taken place; but upon making an examination of the bone of the opposite side, a deformity of the same character, but much less in degree, was found there also.

FIG. 2.

The neck of the specimen shown is depressed so as to form with the shaft below an angle of 45°, much shortened, and increased in its antero-posterior diameter. The head is somewhat enlarged, spread out, and rendered irregular at its junction with the neck by the deposit of little nodules of new bone. The acetabulum is flattened out in correspondence with the increased size and flattening out of the head.

Dr. Richardson supposed the deformity to be the result of rickets, although upon examining the remainder of the skeleton he could find no want of symmetry in the bones except in those connected with the coxo-femoral articulations.

DR. MORTON then read, for DR. R. P. HARRIS, of Philadelphia, a paper on a case of Fallopian Pregnancy, terminating in death.

WEDNESDAY EVENING, December 9th, 1857.

The President in the chair.

*Apoplexy of the Lung, with Disease of the Mitral Valve.*—DR. DA COSTA exhibited a specimen of apoplexy of the lung, accompanying a narrowing of the mitral orifice. T. B., the patient from whom the specimen was obtained, a man thirty-five years of age, came under his care eight months ago, complaining of palpitation of the heart and occasional attacks of bronchitis. A year ago he had suffered from metallic poisoning, which brought in its train a fearful attack of colic, and left him subject to obstinate constipation. Previously, he had had secondary syphilis. At what time he first noticed the irregularity of the heart's beat, he could not remember; he complained of it while being treated for syphilis, but it is very probable that it had at times annoyed him before this period. When first seen, he looked sallow, was troubled occasionally with shortness of breath and palpitation, but was otherwise enjoying tolerably good health. A physical examination of his heart showed the dullness over the precordial region to be increased. The impulse was rather strong, and extremely irregular. A blowing sound was perceptible near the apex; none over the aortic or tricuspid cartilages. The great irregularity and rapidity of the beat rendered it impossible to ascertain whether the blowing sound was synchronous with the systole or diastole; it certainly did not occur with both. The pulse was small, frequent, and intermittent.

He continued in this state until within the last three weeks. His heart beat very irregularly; occasional difficulty in breathing, although this was by no means at any time a very severe or permanent symptom; occasional bronchitis; no dropsy. The sounds over the heart became more and more difficult to analyze, the blowing sound being by no means distinct; in fact, seeming to disappear altogether, while a peculiar clack took its place, or at any rate permitted it but rarely to be heard. This clack was best heard at the apex, faintly transmitted to other portions of the heart, yet sufficiently to obscure the normal sounds; it occurred with but one sound of the heart; with which, could not be determined.

Latterly, he had considerable cough, with frothy expectoration; dyspnœa at night; his face and feet had commenced to swell; diuretics had little influence over the watery effusion.

December 3d.—His symptoms grew much worse. His cough was very troublesome. Large and middling-sized moist râles were heard over his chest. He expectorated considerably. The sputum was not very thick.

During the night of December 6th he was seized with great difficulty of breathing, and pain in his chest. The pain was very severe and sharp on the right side, about the fourth rib. He expectorated a very small quantity of blood.

December 7th.—The pain had much lessened; there were no physical signs over its seat, except the moist râles, which were heard over the greater portion of the chest; the difficulty of breathing continued unabated to his death, December 8th.

Post-mortem examination.—The pleural membrane was perfectly healthy. The lungs were very vascular. In their upper lobes were several dense circumscribed spots, some of a dark, dull color, firm, and with signs of inflammation both in them and immediately around them. This was especially the case with the spot on the right side, corresponding to the seat of pain. Other spots, especially one on the left side, about an inch and a quarter in diameter, contained dark fluid blood, readily scraped away. The effusion seemed, to a great extent, to have taken place into the air-vesicles and minute bronchi.

The heart was enlarged; the cavities of the ventricles somewhat dilated; the walls increased, but not to a very marked extent, and more so on the left side than on the right; the auricle of the left side was considerably distended; its walls very slightly hypertrophied. The tricuspid and pulmonary valves were healthy; so were the aortic, save a trifling thickening, which, however, could not have impaired their function. The mitral valves were extremely diseased. They had been converted into a calcareo-osseous mass, which nearly closed the orifice, leaving an irregular and extremely narrow fissure for the blood to pass through, certainly not the twelfth of an inch in size.

DR. DA COSTA then spoke of the almost entire absence of external hemorrhage in this case.

DR. MOREHOUSE inquired whether the absence of hæmoptysis was not the rule in pulmonary apoplexy? He had seen several cases of pulmonary apoplexy with but trifling, if any, external hemorrhage. In one case, there was diffuse hemorrhage into the cellular tissue of the lung; in another, the effusion of blood into the lung was more circumscribed, and had taken place into the cells alone. Dr. Morehouse also directed attention to the evident signs of inflammation which two of the apoplectic lobules on the table exhibited, and suggested that these might have been the seat of an earlier apoplexy; those in which the blood was dark, and still half-fluid, being of later occurrence.

DR. DA COSTA stated, in reply, that he did not believe that pulmonary apoplexy was either of necessity produced, or connected with, external pulmonary hemorrhage. Indeed, he thought that even where this occurred, the quantity of blood expectorated was usually small. He was able to add another marked case to those mentioned by Dr. Morehouse. The patient, a laboring man, having an enlargement of the heart, was suddenly seized, after violent exertion, with palpitation of the heart, and great distress of breathing, caused, as was afterward ascertained, by nodular apoplexy of the lung. The sputum was merely very slightly tinged with blood. With regard to the signs of inflammation in the specimen before him, he remembered no means of ascertaining, as far as the symptoms went, if the hemorrhage into the lung had occurred at different times. He conceived the inflammation to have been produced by the blood acting as a foreign body.

DR. KEATING thought it a rare occurrence to have hemorrhage from the lung from mere congestion produced by disease of the heart. He mentioned several cases of supposed cardiac hæmoptysis, in which the lungs were found filled with tubercles, which had been the true cause of the hemorrhage, and inclined to the belief that these cases were more frequent than it was supposed.

DR. MOREHOUSE, and several other members of the Society, cited cases of hæmoptysis entirely unconnected with pulmonary disease, and evidently dependent upon the obstructed state of the circulation produced by cardiac disease.

*Urinary Calculus.*—DR. FORBES exhibited a specimen of urinary calculus, taken after death from the bladder of a man aged seventy. It weighs 726 grains. The bladder was sacciform, and much thickened; the calculus was of the mulberry variety, and of a dark color. The rectum presented a fistula in ano; the rest of the organs seemed healthy.

DR. MITCHELL remarked, with reference to the deep dark color of the calculus, that the oxalate of lime, when present in the urine, or in forming calculi, irritates and produces a bloody effusion which frequently tinges the stone, and causes it to contain much iron. Dr. Mitchell had found iron in a large number of urinary calculi—and indeed a little iron is always present in healthy urine, which would be increased if blood were effused. He had examined stones with regard to other metals, especially copper, which he had found in biliary, but not in urinary calculi.

*Cancerous Stricture of the Ileo-cæcal Valve.*—DR. KELLER exhibited a cancerous stricture of the ileo-cæcal valve, removed from a man who had long been subject to obstinate constipation. The stools were clay-colored; only about one passage in ten days. This, with a gradual falling off in health, and latterly with severe pain in the right iliac fossa, and vomiting, were the prominent symptoms. The liver, on post-mortem examination, was found to be healthy; so were the other abdominal viscera. The fæces in the upper bowels were of a green color. The ileo-cæcal valve was converted into a hard cancerous stricture, which presented elements like those of cancer under the microscope. The muscular coat of the intestines was greatly hypertrophied.

DR. LA ROCHE inquired whether the patient had suffered from jaundice.

DR. KELLER stated that he had not. He believed the liver to have been performing its functions properly, and its ducts to have been pervious. The fæces must have lost their greenish color in descending the small, to reach the large intestine.

*Apoplexy of the Brain, consequent upon the use of the Trephine for the Removal of a depressed portion of Bone.*—DR. GROSS exhibited portions of brain and dura mater, removed from the body of a man aged thirty-three. The following was the history of the case:—

G. W. Mason, aged thirty-three, became a patient at the Clinic of the Jefferson Medical College, in November, 1857, on account of a depression of the skull, caused, when he was a boy eight years old, by a blow from a circus pole. The depression was situated on the left side of the head, over the upper portion of the parietal and frontal bones, and was nearly two inches in diameter, by upward of half an inch in depth at its centre. Upon recovering from the immediate effects of the injury, Mason's health remained good until the age of twenty-two, when he was seized with epileptic convulsions, which continued to recur with more or less frequency and severity up to the time of his death. Within the last few months they had assumed so aggravated a character as to disqualify him, in great measure, from following his occupation, which was that of a clerk, his memory having become much impaired, his articulation defective, and his general health much disordered. Under the judicious advice of Dr. Oliver, his family physician, he had made use of a mild alterative course of treatment and a regulated diet, but with no improvement whatever in his condition.

On Wednesday, Dec. 2d, Dr. Gross removed, in presence of the medical class, two discs of bone at the site of the depression, the patient being under the influence of chloroform. The affected bone was remarkably thin, as well as very hard; and when the crown of the trephine had nearly cut its way through, it suddenly caved in, followed by a copious escape of serous fluid. One of the pieces had a long, narrow exostosis upon its inner surface, exhibiting some resemblance to the temporal bone of the fœtus. Corresponding to this projection was a slit-like opening, with well-defined edges, in the dura mater, large enough to admit the point of the index finger. After all the depressed bone was removed, it was ascertained that the dura mater had long been detached for some distance around, its surface having acquired a whitish polished aspect, and formed, along with the inner surface of the bone, a sort of reservoir for the lodgment of the serum which escaped so freely during the operation.

For the first forty-eight hours he appeared to be doing extremely well, although during the first night he had a slight convulsion. At the end of this period he became somewhat stupid, and answered foolishly, but was always able to protrude his tongue. On Saturday he had symptoms of drowsiness, which increased on the following day, when he had also a pretty severe convulsion. He now became soporose, and, although he could be roused, it was impossible to keep him awake longer than a few seconds at a time; he was still able to swallow. On Monday morning, December 7th, he was breathing stertorously, and was evidently in an apoplectic condition. Death occurred at eight o'clock A.M., nearly five days after the operation.

This case, which, so far as Dr. Gross is aware, is unique in its character, is of great interest in a physiological point of view, as showing the importance of the cerebro-spinal liquid in maintaining the functions of the brain, and, especially, the balance of the circulation. Had the membranes of the brain been free from any abnormal opening, permitting the escape of this fluid, it is difficult to conceive how so great an effusion of blood could have taken place after such an operation, as was found in the substance of the brain and its ventricles. Inflammation had certainly nothing to do with the fatal result, since there was no evidence of its existence, either during life or after death.

Thus it will be perceived that the immediate cause of death was apoplexy, occasioned by the removal of the pressure from the brain in consequence of the escape of the cephalo-spinal fluid, destroying the balance of the circulation, and rupturing the cerebral vessels just below the former site of the depression in the skull, where, as was previously stated, the cerebral substance had undergone chronic softening. How long the softening had existed must be a matter of conjecture; but the period must have been considerable, as is proved by the fact that there were old organized apoplectic depôts at the seat of the disease. The last and fatal attack of apoplexy probably began on the third day after the operation, and gradually proceeded, until it rendered the patient completely insensible nearly twenty-four hours before death.

An examination of the body was made twenty-nine hours after death, with the assistance of Drs. Gibbons, Asch, and S. W. Gross. The edges of the wound had partially united. There was no blood or pus between the scalp and dura mater, except a small quantity of semi-organized blood on the membrane, which caused it to adhere pretty firmly to the scalp. The opening in the dura mater, which was opposite the exostosis on the second disc of bone, was well defined, and large enough to receive the end of the index finger. The opening extended also through the arachnoid and pia mater, which accounted for the escape of the cephalo-spinal fluid after the operation, as well as at the time of its performance. On the surface of the brain, below the site of the operation, there was a patch of lymph in the sub-arachnoid areolar tissue. The pia mater was exceedingly vascular. The inflammation of the arachnoid was in itself insufficient to have produced any serious consequences. There were old apoplectic depôts in the furrows of the hemisphere, just below the site of the depressed bone. Upon making a horizontal section of the left lateral lobe, an enormous effusion of black blood was found a short distance below the surface of the brain. This was partly fluid, but for the most part coagulated, and had, as its boundaries, softened cerebral substance, looking very much like spoiled lymph and pus. The cerebral substance in its neighborhood was, in some parts, soft; in others, hardened. The extravasation extended into the middle lobe, in which was also found an old apoplectic depôt, or a semi-organized clot, of a roundish shape and as large as a marble. The left lateral ventricle was filled with blood, a large solid mass extending into its posterior horn, as well as into the third ventricle.

Dr. La Roche did not feel satisfied that the apoplectic clot and softening were recent. The latter especially, he thought, must have existed prior to the operation. He inquired of Dr. Gross whether any symptoms of compression, or of paralysis, had been observed at any time before the operation.

Dr. Gross.—None.

*Tubercle of Lung simulating Cancer.*—Dr. Morton exhibited a specimen of tubercle of the lung, having the exact appearance of cancerous deposits. The lung was filled with hard, roundish masses of various size. When cut into, these did not show any fibrous arrangement, nor were they as hard at the centre as at the periphery.

Dr. Da Costa mentioned that, at the request of Dr. Morton, he had examined these little tumors with the microscope; and although, from their appearance to the naked eye, he had expected to find in them the elements met with in fibroid or in cancerous tumors, yet such was at no portion the case. The round masses exhibited: lung structure much obscured by granules, and on a delicate basement, between the fibrous walls of the air-vesicles, granules, and non-nucleated cells, irregular in shape, small, but in close contact. The cells floating under the field of the microscope were epithelial cells, somewhat shrivelled, a few filled with small oil-drops; and irregular corpuscles, such as are usually designated as tubercle corpuscles.

Dr. Levick stated that it was his opinion that the masses were tubercular. Several of them were softened in the centre. Dr. Levick spoke of the value of minute examination in similar cases. May not some of the reported cases of cancer of the lung and tubercle in other parts of the body be like this? Dr. Levick then commented on the rarity of primary cancer of the lung.

*Enlarged Vermiform Appendix.*—DR. MORTON next exhibited an enlarged vermiform appendix, nine inches in length, and filled with fæcal matter. It had not, during life, given rise to any symptoms.

---

WEDNESDAY EVENING, Dec. 23d, 1857.

The President in the chair.

*Cancer of Several Organs.*—DR. MORTON directed the attention of the Society to the cancerous viscera on the table. The history of the case is as follows:—

"Margaret Brown, aged thirty-five, a native of Ireland, was admitted into the Pennsylvania Hospital, November 25th, 1857, on account of a very painful tumor on the surface of the abdomen, at the base of the sternum; it had quite a carbuncular appearance. She stated that the tumor first showed itself about four months before her admittance, and that it came as a small, hard, round nodule, which gradually increased in size; it had a very hard base, and was softer toward the middle. She has always had a great deal of pain in it, more of late, and most during the night. She said her bowels had always been costive. She was nursing an infant, five months old, when she came into the hospital, but her milk failed her about two weeks before her death; her health had been very good until within a short time, and she had never any pains in other parts of her body. She was put upon a tonic treatment; and the tumor, which seemed as if it contained pus, was poulticed, and opened and discharged a thin watery matter. On the morning of the 20th of December she complained of a soreness in her neck; marked symptoms of tetanus set in, which terminated fatally at five P.M. the next day.

Post-mortem examination fourteen hours after death.—The tumor on the abdomen extended only through the parietes, and was attached to the cartilages of the ribs and sternum; when cut into, it presented a cancerous appearance. The lungs and heart were healthy. The kidneys were full of cancer-spots all over the surface; one cancerous mass was found in the cellular tissue, near the pelvis of the left kidney; a large cancerous tumor was found occupying the right side of the pelvis, over the sacro-iliac junction, as large as two fists; through it the iliac vessels and nerves passed, entirely surrounded by the tumor. A small mass was found in the left axilla, which had the same characteristics as the other tumors. Other organs healthy. Portions of each tumor were examined under the microscope, and exhibited cancer-cells.

DR. DARRACH suggested that the lesion of nerves which were involved in the tumor near the ileum might have been the cause of the tetanus.

*Hairy Mole on the Cornea of an Ox.*—DR. DARRACH exhibited a hairy mole on the cornea of an ox. The ox, during life, had not its sight affected by the mole, and could close the lids without difficulty. The surrounding cornea was healthy.

The mole was situated on the upper and outer portion of the cornea; was attached to the conjunctiva by a band of conjunctival tissue; it had a broad base, and was covered with hairs from six to eight lines in length. The mole at the base was about five and a half lines, and about five in height.

DR. HEWSON stated that a patient at Wills Hospital had been lately under his care who had a wart on the inner border of the cornea similar to the one exhibited. It was not, however, covered with hair.

*Large Gall-stones; some impacted in Hepatic Ducts.*—DR. PACKARD exhibited a gall-stone, oval, concentric, with rings of darker and lighter brown; 1 inch and $\frac{9}{16}$ in length, $\frac{15}{16}$ in width, (measuring the section;) it had completely distended the gall-bladder. The liver was natural in appearance, but in the left lobe the hepatic ducts were found distended to the size of the portal vein by gall-stones of various sizes, all soft and brown.

No other visceral disease existed. The patient was a sailor, who had fallen overboard from a vessel, fracturing his right thigh, and remaining in the water about an hour; the accident occurred about four days previous to his death, and no reaction took place in the interval.

DR. HALL exhibited the four extremities of a woman who died of idiopathic gangrene, while he was resident in the Episcopal Hospital. The case was, at the time, fully reported in the March number of the *Medical Examiner*, 1856.

*Vesical Calculus.*—DR. MITCHELL exhibited a mace-shaped calculus. This curious stone (Fig. 3.) was removed post-mortem, by Dr. Pancoast,

FIG. 3.

Outside.—Nat. Size. Section magnified 2½ or 3 diameters.

from the bladder of Mr. B. Its form is well enough illustrated by the plate, where the flat surface of the section is seen enlarged, the rough outside being drawn of the natural size. The dark portion is prolonged from the centre into ray-like projections, and is, like them, composed of oxalate of lime. The intervening white substance is phosphate of ammonia and magnesia, with phosphate of lime. Two calculi resembling this one are described in the *Amer. Journ. of Med. Sci.*, vol. iv. p. 333. The present specimen is certainly a pathological curiosity, for whose singular form we are quite at a loss to account.

DR. DARRACH, in commenting on the different forms assumed by urinary calculi, mentioned that an organic mould had been discovered to be the ground-work of many; the salts being deposited around. This mould had, at least on a small scale, been lately produced artificially.

DR. MITCHELL spoke on the same subject, and stated further, that it was the belief of some of the French observers that even separate crystals had an organic stroma.

*Necrosis of Head and Upper Part of Os Femoris removed by an Operation of Excision.*—DR. ADDINELL HEWSON exhibited a specimen of necrosis of the head, neck, and portion of the shaft of the left os femoris, which he had removed the week previous, by excision, from the hip of a girl aged thirteen years. The girl had suffered with disease in her hip for more than two years, but had had no care whatever bestowed upon her during that time. She had been living on a truck-farm, near the city, where her father was but a common day-laborer. She had lost her mother seven years before by phthisis. Soon after the first appearance of the lameness in the joint an abscess formed and slowly worked its way to the front of the thigh, where it opened, about three inches below the groin. This opening shortly closed, and the head of the bone became dislocated on the dorsum ilii, and she walked about by leaning forward and resting on the ball of the foot. In the course of three or four months the matter reaccumulated and discharged itself through the old opening. About ten months since an abscess formed on the inside of the thigh; it grew but slowly, and was finally opened by the patient herself, with a needle, some three months before her admission into the Wills Hospital.

When she was admitted (Dec. 2d) this opening was still discharging a thin sanies, and on the back of her thigh, just at the lower border of the buttock, there was a large purulent collection. The integuments were here discolored to the extent of four or five inches in circumference, and the tumor beneath was large and fluctuating. The thigh was flexed almost at a right angle to the pelvis, and admitted of but little motion, although it was not firmly anchylosed. No crepitation or grating could be elicited.

Her health had been failing her for the last five months; her belly was tumid, but her bowels were regular, and their dejections natural. She was excessively pale and emaciated, and suffering from hectic. Her pulse was 130, feeble and flickering. There were no signs of pulmonary or other organic disease. She had acquired great mobility in the lumbar vertebræ by her efforts to hold herself erect.

The abscess in the region of the buttock was opened a few days after her admission into the hospital, and from it there were discharged about two ounces of fetid pus. No direct communication to the head of the bone could be detected through this abscess, nor was there any dead or exposed bone detected by the probe. After the tumefaction, however, had subsided, the pus could be made to flow out of the opening by pressure above the head of the bone, showing clearly that this abscess was connected with the joint.

The operation of excision was performed December 16th, and the head, neck, and three and a half inches of the shaft (measuring from the point of the great trochanter) were removed through a T incision, made over the joint on its outer side. The head of the bone was found lying just above and behind the edge of the acetabulum, where it had formed a deep cup of bone for itself. Both the head of the bone and this new acetabulum were profoundly necrosed. The old cavity was partially filled up with organized lymph, and entirely free of disease. The head and neck of the bone, necrosed through their whole thickness, had partially undergone molecular exfoliation. They were much reduced in size. The portion of the shaft removed in the operation was entirely denuded of its periosteum, saving the great trochanter, on which this tissue was dense and firm, and somewhat thickened. No exfoliation had occurred in the shaft. Here the necrosis appeared to be recent, and to involve only the external lamella of bone. The periosteal covering of this portion was almost entire, although separated by purulent matter from the bone.

Dr. Hewson remarked that the specimen illustrated very admirably, he thought, the progress of *morbus coxarius* in its advanced stages, and how very ineffectual the unaided efforts of nature must sometimes be to cure the disease. Here had evidently been disease involving only the fibrous structures of the joint in the first instance, or, if the disease was more profound, it affected only the surface of the head of the thigh bone, but not the acetabulum also. Suppuration ensued, and the capsule of the joint giving way, the head of the bone became dislocated on to the dorsum ilii. Here nature attempted a cure—a new cavity was formed to receive the bone—but sufficient time was not allowed for the repair to be perfected. The little patient not being restrained, used the limb too soon and too much, and so re-established the destructive action, or, perhaps, rather excited that which was latent, or had nearly subsided. But now the morbid process extended beyond its original limits. It now not only involved the head of the bone, but had attacked the new cavity formed for its reception. Necrosis of both these parts was the result, and nature's efforts to get rid of the dead tissue only involved a further destruction of the thigh-bone; for the pus formed in the process of *insensible exfoliation* which ensued, burrowed down along the neck and shaft of the bone as along a director, denuding those parts, and consequently causing their ultimate death. To what extent this destruction would have gone, it is impossible to say; but it must be evident to all who have seen the patient, that she would have sunk under exhaustion long before nature could have effected the separation of such a mass of dead tissue, even if she could have done so without further expense to the living bone.

The patient is now doing well, and the cure in her case is steadily progressing.

DR. HARTSHORNE, who was present at the operation, made some remarks on the state of the cavities in which the head of the bone had rested. The old was partially filled up; the new, it had appeared to him, communicated with this by means of a narrow passage.

*Bright's Disease.*—DR. HUMPHREYS exhibited a specimen of Bright's kidney, small, granular, and hard. No history attached. The patient was admitted for an injury, into the Pennsylvania Hospital.

DR. LEVICK alluded to the similarity of this kidney with the one described as the "gouty kidney."

DR. DARRACH spoke of the different forms of chronic Bright's disease, and the resemblance between them and some morbid states of the liver.

About four or five years ago, while resident physician at the Pennsylvania Hospital, his attention was attracted to the analogy existing between the diseases of the kidney and the liver, by the observation, at post-mortem examinations of these organs. Perhaps the best method to present the subject will be to briefly mention illustrative cases.

CASE I. was a granular kidney, small in size, consistence firm, much granulated, surface and outline very irregular, with thickening and solidification of the fibrous tissue about the hilus. Here we have an affection closely resembling in appearance that which has been termed cirrhosis of the liver, and although the fibrous tissue at the hilus of the kidney cannot be considered as analogous in function to that of the capsule of Glisson, yet in structure it is the same; and they are further alike that it is the fibrous tissue of the substance of the kidney which is the seat of inflammation and deposit in this form of kidney disease, causing thus shrinking and lobulation, or granulation. This, then, is the point to which Dr. Darrach would attract attention, that it is in this form of kidney affection we find the fibrous tissue of the organ much affected. This disease is quite distinct from the large white kidney, and not a more advanced stage of the same affection.

CASE II. The kidney was rather above normal size, consistence firm, smooth on the surface, and pale, and when it was laid open with the knife the cut surface appeared like wax (the demarcation between cortical and pyramidal portions barely discernible) of a uniform yellowish white color, smooth and polished.

This form certainly bears a close resemblance to the lardaceous (bacony) liver; the oily or fatty kidney is also an affection which is too notably allied to oily liver not to be here mentioned.

The more distinct forms of *chronic* kidney affection, commonly classed under the designation of Bright's disease, which have come under Dr. Darrach's observation, are—1st. The lobulated granular kidney, becoming in the last stage very small; met with in persons not especially addicted to drink, nor of a scrofulous nature. The case above referred to having occurred in a man who was a farmer, of good habits, but who had been much exposed to epidemic and endemic influences, having suffered from intermittent and remittent fever. The 2d forms are—*a.* Large yellowish-white kidney, with cortical and pyramidal portions sufficiently distinctly marked, often with yellowish deposits of granular matter in the neighborhood, or in the corpuscles, sometimes having the pyramidal portion reddened, while the interval is of a yellowish color, and again with both portions of much the same hue; *b.* The albuminous, waxy, homogenous kidney. 3d. The oily kidney, which Dr. Darrach has noticed more frequently than in the other cases, accompanied with a corresponding condition of liver.

DR. WOODWARD mentioned, regarding the fatty kidney, that he had frequently seen it unaccompanied by albumen in the urine In twelve cases of fatty degeneration of the kidney, albumen was only twice or three times present.

---

WEDNESDAY EVENING, Jan. 13th, 1858.

The President, DR. GROSS, and afterward Vice-President, DR. LA ROCHE, in the chair.

*Cicatrix and Chalky Formations in the Lungs.*—DR. LEVICK exhibited a specimen of cicatrix and chalky formations in the lungs, obtained from a patient aged eighty-two, whom he saw, with Dr. Stroud, at the Widow's Asylum. It was not known that the patient had ever had phthisis. She was latterly very weak and much emaciated, and the impulse of her heart was not distinct; but she died without presenting any remarkable symptoms.

Post-mortem examination.—The heart was found to be very small; it resembled a case of concentric hypertrophy seen by Dr. Levick some time ago; its walls being somewhat thick compared with the size of the cavities. The valves were healthy, excepting the aortic valves, which were slightly thickened by a deposit. A calcareous and atheromatous deposit was observed along the aorta, but did not extend into the large vessels. The arch of the aorta seemed rather dilated. The spleen was very small, firm, and covered with a fibroid deposit. The lungs were of a dark color; at the upper portion of each several calcareous bodies were met with. At the apex of the left lung a distinct cicatrix existed. Dr. Levick had no doubt that the little masses were tubercles which had undergone a calcareous degeneration, and thus the patient recovered.

DR. LEVICK then exhibited a specimen of general melanosis, which had been presented at the last meeting of the College of Physicians. The patient died of cancer of the pylorus. Melanotic masses were found in the lung, bronchial glands, liver, kidney, stomach, lips of the womb, intestines, and gall-bladder.

Dr. Levick spoke of the general tendency to pigmentary deposit in this case.

DR. MITCHELL stated that he had examined a small portion with the microscope, and found cancerous elements and numerous pigment masses and granules.

A discussion arose as to the connection of cancer with melanosis, whether pigment masses might not be extensively deposited without cancer.

DR. WOODWARD believed that they might, although they usually occur associated with it.

DR. STILLÉ thought that the evidence here was strongly in favor of cancer, as there was cancer of the stomach, and as evidently the bulk of many of the masses was not made up of pure melanotic matter, but of a whitish, firm substance.

*Urinary Calculus.*—DR. DARRACH presented a specimen, belonging to DR. PEPPER, of an oxalate of lime calculus, with projections, similar to the one exhibited at the last meeting, (see Fig. 4.) It was removed after death from a man, eighty years of age, along with several smaller ones. The drawing will give a better idea of its form than any written description can. The smaller calculi were of a yellowish-gray color, smooth, except at points; where there could be perceived, in some quite manifest, in others less so, the same disposition to become bossed as in the larger calculus. All the history which could be obtained of the case is, that the patient suffered very much, and a short time before death had a great deal of pain, which he thought was owing to retention

FIG. 4.

of urine. The physicans were unable to introduce a catheter, and after death the bladder was found about the size of the fist, and its walls much thickened.

DR. DARRACH did not believe the calculus had been formed around a foreign body.

*Cancer of Cicatrix.*—DR. ADDINELL HEWSON exhibited a specimen of malignant fungus, which attacked the stump of a thigh amputated by him some seven months previous, for a large encephaloid tumor growing from the periostium of the lower portion of the os femoris.

The disease had occurred in a lady, aged thirty-eight years, of marked tubercular tendency, and in whose family cancer had never before made its appearance. Most of the immediate family, except her parents, had died of phthisis. Both of her parents lived to an advanced age, and died of diseases peculiarly incident to that period of life. She had early in life several attacks of hœmoptysis. On the first appearance of the disease the symptoms of its malignancy were exceedingly tardy in manifesting themselves; for although she complained of pain in the limb for nearly ten months before it was amputated, it was really not until within about ten days of the time of the operation that a positive diagnosis of the nature of the disease was arrived at. This arose from the previous history of the patient: her appearance and her uncomplaining disposition. She was, as before stated, of a phthisical tendency, but appeared in good health, with a bright, clear complexion. The pain with which she suffered she complained of only occasionally, although she afterward confessed that it had been very constant. This pain was aggravated at night, and increased by pressure, but not by jarring the limb. The thigh continued but little swollen for a long time after the first manifestation of the pain, and there was no venous enlargement or discoloration and glazing of the skin until within a short time of the operation. Her case was, therefore, viewed for a long time as one of idiopathic periostitis, and treated as such; but as it progressed, as the limb became enlarged, the pain more vivid, and the general health impaired, in spite of the treatment resorted to for relief, the accuracy of the previous diagnosis became doubtful, and after several consultations and an exploration with a fine exploring trocar, the conclusion was arrived at that the disease was malignant. The use of the exploring needle really revealed nothing of itself, but greatly aggravated the disease. Previous to its use the tumor was increasing but slowly; after the exploration it grew so as to add half an inch in circumference to the limb in every twenty-four hours. Amputation was performed on the 2d of July, ten days after this exploration. The thigh was removed by the double flap operation, as high up as possible, with the tourniquet applied, and so loaded was the limb with adeps that a catlin nine inches long was just sufficient to transfix it; this was somewhat remarkable, as the patient was neither large nor fat in person. A large amount of blood was lost during the operation, owing to the compression by the tourniquet not being sufficient to arrest the circulation. The tumor proved to be an encephaloid mass the size of a large fist, attached to the periosteum; the bone had been denuded in one place by its pressure. The portion of the tumor which had been penetrated by the exploring needle was very vascular, and presented a marked contrast with the brain-like color and appearance of the rest. Although some erysipelas followed where the skin had been bruised in the efforts to make the tourniquet arrest the hemorrhage, everything did well for a time; the wound all healed up except a little point through which the femoral ligature had been discharged, and the patient was out and riding about, with a pulse between 80 and 90. So matters continued, until about three months after the amputation, when symptoms of a return of the disease showed themselves in the cicatrix. These became more prominent, and the whole scar became gradually involved in large fungus excrescences, which bled freely at times. The patient finally died, exhausted by the disease. Her suffering, however, was at no time after the operation ever so great as it was before.

The specimen exhibited the fungus disease involving the cicatrix through its whole depth. There were no signs of the disease having attacked the bone, which was exhibited with its edge well rounded off. It was, however, found, after sawing it through, to be very vascular. A remarkable circumstance noticed at the autopsy was that the adipose tissue of the stump was as thick as on the day of the operation, although the patient had become emaciated to an extreme degree before her death. This tissue was still two inches in thickness; the other tissues were all wasted away, so that the stump really seemed to consist merely of the bone, adipose tissue, skin, and malignant disease.

*Exostosis of Scapula.*—DR. GROSS exhibited, for DR. KERR, of China, a specimen of exostosis of the scapula, removed from a laboring man, aged sixty years, who was admitted into the Ophthalmic Hospital in Canton, in the summer of 1855, with a bony growth from the scapula, which projected about an inch and a half through the skin, giving rise to a constant discharge of pus. The tumor was attached to the inner border of the scapula, half way between the spine and the inferior angle. Its shape was irregularly cylindrical, three inches long, with a diameter of an inch and a half. One end extended half an inch below the under surface of the scapula, raising this bone that much above its ordinary level; under the end which rested on the ribs a bursa mucosa was developed. So little of the scapula was involved that it was only necessary to saw out a piece two and a half inches long by three-fourths of an inch wide. The patient sank from the effects of the operation, and died on the fifth or sixth day.

*Enlargement and Induration of the Pancreas.*—DR. STILLÉ exhibited an enlarged and hardened pancreas, taken from a patient aged sixty, whom he had seen in consultation, and who for two years had been suffering from dyspepsia, acid eructations, very dry mouth, deep-seated and dull pain at the epigastrium, vomiting of food, gradual loss of flesh, and during the last six months diarrhœa. No pain in lower part of abdomen. Urine free and appeared normal. During two months or more before death there was gradual emaciation and loss of strength; at one time purpura, which disappeared under the use of mineral acids and iron. Stools of the consistence and color of Indian-meal mush, slimy, and at times three or four a day in number; but generally, under the use of astringents, not more than two in three days. They presented no evidence of containing fat. No tumor of abdomen, nor tenderness of epigastrium, where deep pressure excited only a dull pain. No pulmonary symptoms of note. Some œdema of hands and feet in the last fortnight of life; death occurred by asthenia. Jaundice, it was stated, had at one time been present, but not as a marked symptom; while under Dr. Stillé's observation there was none.

Post-mortem examination.—No visible disease of exterior of bowels. Liver of normal color and size. No examination of viscera was made except of the pyloric end of stomach and of the pancreas; the former was healthy. Pancreas enlarged one-third in all dimensions; hardened. Under the microscope it showed a great increase of fibrous tissue, and in parts a considerable amount of fat.

DR. STILLÉ would not assert that the symptoms arose entirely from the pancreatic disease, particularly since Handfield Jones, after analyzing thirty cases of diseases of this viscus, renders it probable that "no symptoms give any intimation of the existence even of the most advanced pancreatic degeneration," and a similar judgment has been expressed by Dr. Chambers.

Dr. Stillé then read an extract from a case published in the *Lancet* of Nov. 10th, 1855, which resembled this in several points. He also alluded to one which he had examined a long time ago, in which acid vomiting, anæmia, with wasting away, were marked symptoms, and in which he thought it probable the symptoms were caused by a chronic disease of the pancreas. In conclusion, Dr. Stillé dwelt on the absence of satisfactory knowledge with regard to the symptoms of pancreatic diseases.

DR. MITCHELL alluded to the experiments of Bernard, who had found an affection of the intestinal villi to follow extirpation of the pancreas.

DR. HARTSHORNE referred to a case of pancreatic disease which had come under his observation many years ago. The symptoms, as far as he could distinctly remember them, were a constant pain in the neighborhood of the epigastrium, dyspepsia, loss of flesh, and vomiting. The man died suddenly, and on a post-mortem examination made with Dr. Van Buren, the pancreas was found enlarged and hardened. Its head was converted into a firm dense tumor.

*Cysts from the Liver Coughed up through the Pulmonary Passages.*—DR. RICHARDSON exhibited, at the request of DR. F. GURNEY SMITH, specimens of cysts from the liver, which had been given to Dr. Smith by a medical gentleman, who had also furnished a brief history of the case:—

Mrs. A., aged thirty-four; married ten years; without children. From girlhood has had symptoms of diseased liver, which, at intervals of one or two years, have produced acute paroxysms of pain and enlargement, that no treatment affected. Last winter was passed on the sea-board, near Boston, with general health good, but growing tenderness at the hypochondriac region; the side swollen even to distortion of the ribs; much pain, and two very tender spots, one in front, the other on the back, nearly opposite. Numbness in the arm and foot of the right side has been a constant symptom, with inability to lie upon that side. Febrile symptoms were occasional; the natural pulse being about 80, would rise to 95; respiration good.

Early in April last the symptoms of disease became more acute. The swelling of the side, pain, and soreness increased rapidly. Great pain in the top of the head, with acceleration of the pulse took place; for five nights she was sleepless, and rest was only finally procured on the sixth day.

Two days after this, without premonitory symptoms of any kind, there was a large discharge from the bowels of fetid, purulent, and bloody matter, which, continuing for two and a half hours, produced great exhaustion; so great that, for several days following reaction seemed doubtful. The stomach was too weak to retain more than a spoonful of even fluid food, and without nausea rejected almost everything but ice-water. This condition continued for about ten days; extreme emaciation followed; numbness of the extremities of the right side; night-sweats and swollen feet. Cough set in, with expectoration of frothy saliva at first, afterward sputa, resembling those of pneumonia. The tongue had a thick, white coat; the pulse rose to 120, feeble and wiry; the stomach was less irritable. By very attentive nursing the patient commenced to improve, and continued to do so for the next two months, though the same symptoms more or less existed. The months of July and August were passed at Center Harbor, N. H., without much further amelioration, and she returned to the neighborhood of Boston by September 1st. About this time cysts were first coughed up, one, two, or three in a day, of the size of a filbert or less, to that of a pea, all much torn. They have been closely examined by more than one observer, but not a trace of parasite has been found. The discharge of cysts became gradually less frequent, but more numerous and more variable in size. Sometimes, after an interval of five days, twenty or thirty even would be thrown off at one paroxysm of coughing, that lasted two or three hours. More recently these paroxysms have occurred once in ten days, and the cysts are discharged during perhaps the whole of two days. Some have been so perfect as to have but one small aperture, and have been inflated by the blow-pipe; some smaller than a pea have come up unbroken, but soon after collapsed. Their contents are apparently purely serous, though the same thick expectoration accompanies them as at first. With few exceptions, the walls of which are translucent, the cysts are opaquely white, and under the microscope exhibit only occasional striæ.

The present condition of the patient is with much less pain and suffering than she had last winter, though with less strength and more difficult respiration. She is able to go out in good weather, and takes the usual exercise in the house. Some numbness of the limbs and considerable soreness of the chest remains. The sputa contain some clots of blood. The paroxysms of coughing are exhausting, violent, and spasmodic, resembling hooping-cough.

DR. HARTSHORNE, in reply to a remark from one of the members of the Society, who considered the symptoms as inconclusive by which the hepatic origin of the cysts was attempted to be proved, supplied, from his knowledge of the case, some facts not dwelt upon in the report, especially with regard to the purulent diarrhœa. He also stated that the patient had been, and was being, narrowly watched by several physicians in Boston, who were satisfied of the hepatic origin of the cysts, and would give to the profession the full particulars of this interesting case.

## Figures and Tables

**Fig. 1. f1:**
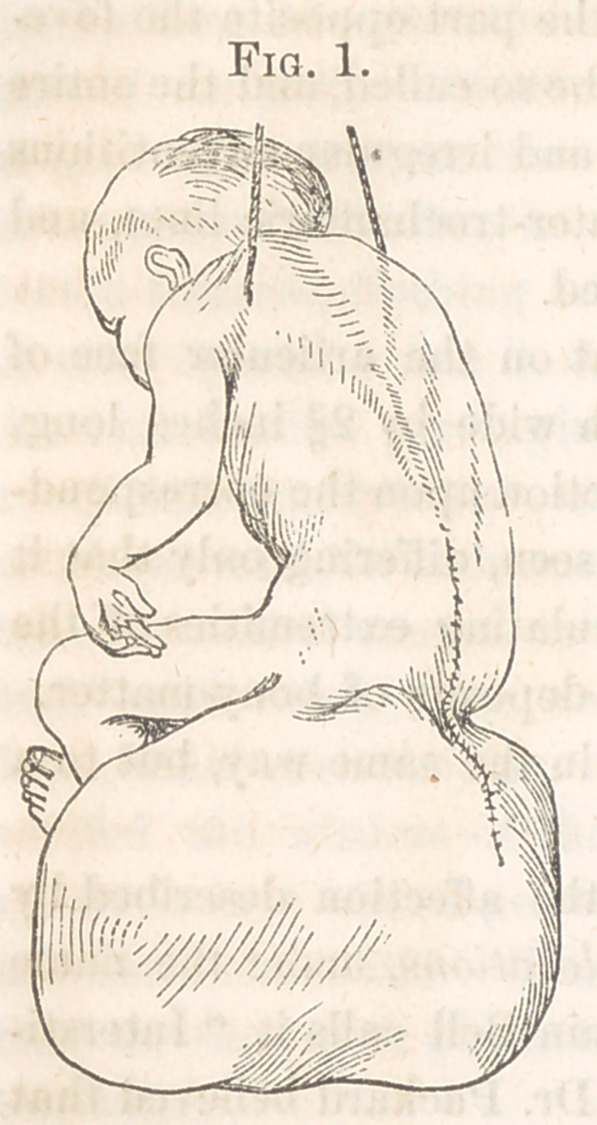


**Fig. 2. f2:**
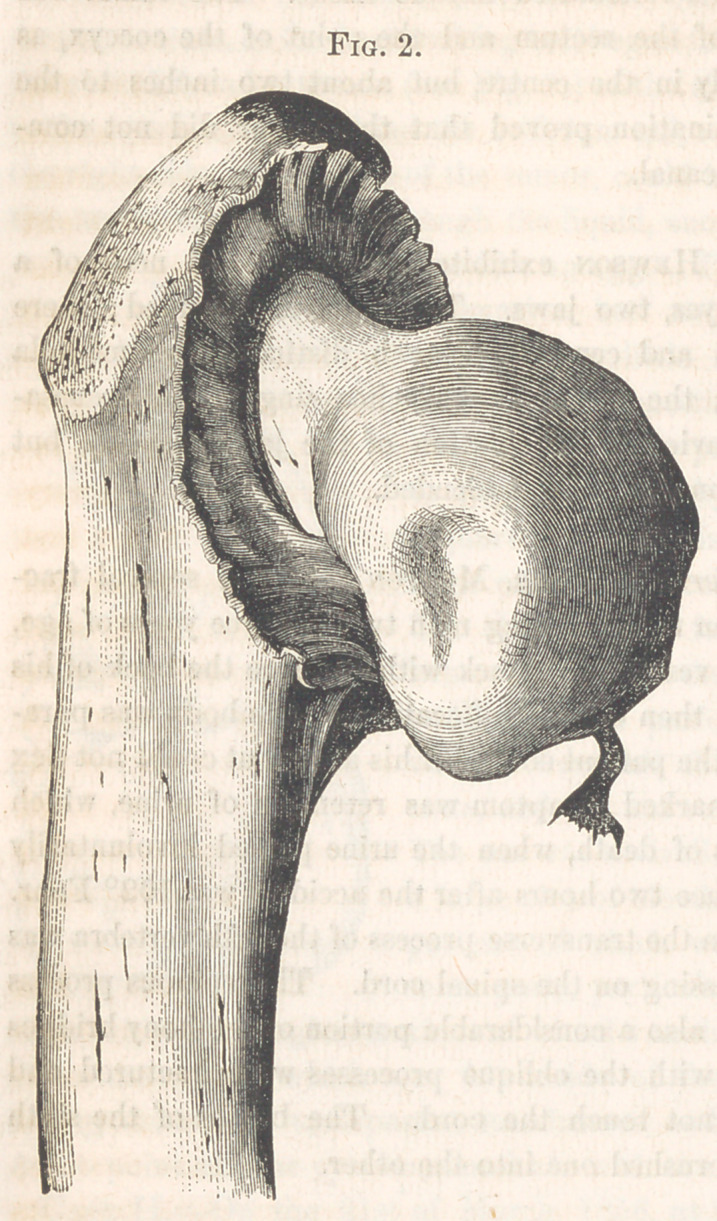


**Fig. 3. f3:**
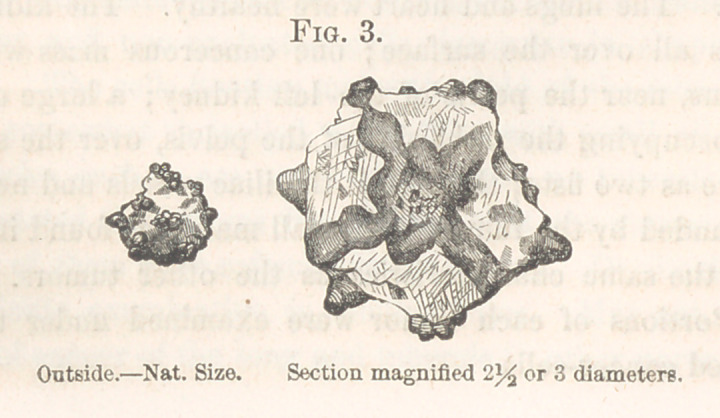


**Fig. 4. f4:**